# The Moderating Role of COMT and BDNF Polymorphisms on Transfer Effects Following Multi- and Single-Domain Cognitive Training Among Community-Dwelling Shanghainese Older Adults

**DOI:** 10.3389/fnagi.2018.00198

**Published:** 2018-07-05

**Authors:** Jiangling Jiang, Alexandra J. Fiocco, Xinyi Cao, Lijuan Jiang, Wei Feng, Yuan Shen, Ting Li, Chunbo Li

**Affiliations:** ^1^Shanghai Key Laboratory of Psychotic Disorders, Shanghai Mental Health Center (SMHC), Shanghai Jiao Tong University School of Medicine, Shanghai, China; ^2^Department of Psychology, Institute for Stress and Wellbeing Research, Ryerson University, Toronto, ON, Canada; ^3^Department of Psychiatry, Tongji Hospital of Tongji University, Shanghai, China; ^4^Department of Psychiatry, Shanghai Tenth People’s Hospital of Tongji University, Shanghai, China; ^5^Shanghai Changning Mental Health Center, Shanghai, China; ^6^Brain Science and Technology Research Center, Shanghai Jiao Tong University, Shanghai, China; ^7^Center for Excellence in Brain Science and Intelligence Technology (CEBSIT), Chinese Academy of Sciences, Shanghai, China

**Keywords:** cognitive training, catechol-O-methyltransferase, brain-derived neurotrophic factor, single nucleotide polymorphism, successful aging

## Abstract

Given the increase in research suggesting benefit following cognitive training in older adults, researchers have started to investigate the potential moderating role of genetic polymorphisms on transfer effects. The objective of this study was to evaluate the moderating effect of catechol-O-methyltransferase (COMT) and brain-derived neurotrophic factor (BDNF) polymorphisms on transfer effects following a single-domain or multi-domain training intervention in healthy community-dwelling older adults. A total of 104 men and women living in Shanghai were randomized to a multi-domain or a single-domain cognitive training (SDCT) group. COMT rs4818 SNP and the BDNF rs6265 SNP were analyzed from blood. At pre-intervention, post-intervention and at 6-month follow-up, participants completed the Repeatable Battery for the Assessment of Neuropsychological Status (RBANS), the Color-Word Stroop Test (CWST), the Trails Making Test (TMT) and the Visual Reasoning Test (VRT). COMT was found to moderate immediate memory transfer effects following single-domain training only, with G/- carriers displaying greater benefits than C/C carriers. BDNF was found to moderate attention and inhibition independent of the training, with Met/- carriers displaying better performance than Val/Val carriers. Overall, individualizing training methods with full consideration of genetic polymorphisms may promote the maximization of cognitive training benefits.

## Introduction

Thanks to the advances in medical technology and health care systems, countries around the globe are experiencing a rise in the older adult population (World Health Organization, 2015). With an increase in life expectancy comes an increased risk for the development of non-communicable diseases, including dementia. Due to the insidious nature of the disease and the lack of promising treatments available, dementia has become one of the most challenging chronic diseases faced by society and the healthcare system (World Health Organization, 2015). Furthermore, in the context of an aging population, the prevalence of dementia is expected to rise exponentially (World Health Organization, 2015). Currently, dementia is estimated to affect approximately 50 million individuals with a cost of more than 600 billion dollars per year across the world (World Health Organization, 2015). Consequently, there is a growing impetus to discover interventions that may delay or prevent disease onset and to delineate the mechanisms that determine intervention efficacy. Cognitive training is one intervention that has received growing interest as a tool to minimize cognitive impairment in late adulthood (Reichman et al., 2010).

Over a decade of research suggests that cognitive training is a viable intervention to enhance cognitive functioning among healthy older adults (Cheng et al., 2012; Lampit et al., 2014), persons with mild cognitive impairment (Li et al., 2011), and persons with dementia (Sitzer et al., 2006). Despite these promising findings, one must consider individual variability in the intervention response and the underlying mechanisms that may explain this variability. Among the potential mechanisms, it is postulated that cognitive training may generate its beneficial effects by upregulating neurotransmitters, such as dopamine (Bäckman et al., 2011), as well as neurotrophins, such as brain derived neurotrophic factor (BDNF; Vinogradov et al., 2009), two neurochemicals that are found to deplete with aging (Komulainen et al., 2008; Tapia-Arancibia et al., 2008; Morcom et al., 2010).

The catechol-O-methyltransferase (COMT) enzyme modulates dopamine levels in the prefrontal cortex by degrading dopamine into 3-methoxytyramine (Bäckman et al., 2006). Consequently, the COMT gene has received attention as a genetic contributor to variations in cognitive function. The gene that codes for COMT contains a functional common Val^158^Met polymorphism, with the wild-type Val allele exhibiting a three- to four-fold increase in enzyme activity, resulting in lower extracellular prefrontal dopamine, compared with the substitution Met allele (Chen Z.-Y. et al., 2004). Several cross-sectional studies in young adults show that the Val genotype is associated with less efficient cognitive processing and poorer performance on cognitive tests compared to the Met/Met genotype (Bruder et al., 2005; Caldú et al., 2007). However, in a prospective study of 2, 857 older biracial men and women aged 70–79, it was found that COMT did not associate with baseline cognitive function. Furthermore, in evaluating cognitive trajectory over 8 years, COMT_Met/Met_ was associated with accelerated decline compared with Met/Val and Val/Val carrier status. Specifically, Met/Met was associated with greater decline in global cognitive function, measured by the Mini-Mental State Examination; and executive function, measured by the Digit Symbol Substitution Test (Fiocco et al., 2010). Although less investigated, other functional polymorphisms in the COMT locus, including the rs4818 (C1886G, Leu136Leu, synonymous) is found to modulate COMT activity, with the C allele variant associated with higher enzymatic activity than the G variant (Diatchenko et al., 2005).

Polymorphism in the BDNF gene, resulting in a Val to Met substitution at position 66 in the prodomain (BDNF_Met_), is significantly associated with reduced BDNF secretion (Chen J. et al., 2004). Although a recent meta-analysis of 21 studies concluded that BDNF Val66Met does not associate with cognitive function, age of the study samples was not considered, which may have affected the meta-analytic outcome (Mandelman and Grigorenko, 2012). Indeed, research suggests age-based differential effects of BDNF polymorphism (Verhagen et al., 2010). While a number of studies in young (Freundlieb et al., 2015; Enge et al., 2016) and middle-aged (De Beaumont et al., 2013) adults suggest that carriers of the Met variant exhibit increased susceptibility for impaired cognitive function, research that focuses on older adult cohorts suggests a differential pattern, with Val/Val carriers displaying increased risk for accelerated cognitive decline (Harris et al., 2006). For example in a 10-year longitudinal study of healthy older adults aged 67–86 years (Erickson et al., 2008), it was found that Val homozygotes displayed greater decline in executive function compared to Met allele carriers over time. The authors postulated that this beneficial cross-over effect may occur in the seventh decade of life (Erickson et al., 2008).

Recently, research has shown a potential role for BDNF (Freundlieb et al., 2015; Enge et al., 2016) and COMT (Panizzutti et al., 2013; Colzato et al., 2014) polymorphisms in moderating the cognitive training response in young adults and clinical populations (i.e., schizophrenia). In the COGITO study (Bellander et al., 2015), which evaluated the transfer effects of cognitive training in 47 young adults and 78 older adults, it was found that homozygote Val allele carriers performed more poorly at baseline but showed greater improvement for working memory near transfer. Although young adults displayed greater gains following cognitive training relative to older adults, analysis did not reliably discern a differential age-effect for the moderating role of COMT on transfer effects following cognitive training (Bellander et al., 2015). While additional research is needed, this study suggests that full consideration of the impact of genetic polymorphisms may provide an opportunity to maximize training benefits in older adults. Accordingly, research is needed to examine genetic correlates of the cognitive training response in older adults.

In a previously reported randomized controlled trial (RCT) by the authors, examining transfer effects following cognitive training in community-dwelling older adults living in Shanghai, it was found that older adults in the single-domain or multi-domain cognitive training (MDCT) program exhibited enhanced post-training cognitive performance, compared with a wait list control group (Cheng et al., 2012). Given the aforementioned associations between cognitive function and biological correlates of brain function, including BDNF and COMT polymorphism, and the potential modulating role of genetic polymorphisms in transfer effects following cognitive training, the objective of this study was to identify differential response patterns to cognitive training that may result from variants of the COMT and BDNF single nucleotide polymorphism (SNP). More specifically, this study explored the moderating role of BDNF and COMT on cognitive transfer effects across and between training type (i.e., single-domain and multi-domain training) in this sample of older adults.

## Materials and Methods

### Participants

A total of 270 healthy Chinese older adults were recruited from the Ganquan-area community in Shanghai. Details of the study design and CONSORT flow diagram are previously reported (Cheng et al., 2012). Briefly, participants were eligible for the study if they were: (1) between 65 years and 75 years of age; and (2) physically able to attend the training courses. Exclusion criteria were: (1) illiteracy; (2) presence of vision, hearing or communication deficits; (3) presence of significant cognitive impairment, measured by the Chinese Mini-mental status exam; (4) functional impairment with difficulties in living independently; and (5) presence of an existing neurodegenerative disorder, major neurological and/or psychiatric disorder (e.g., stroke, depression, or schizophrenia), or current diagnosis of cancer.

### Study Design

This study was carried out in accordance with the recommendations of the Human Research Ethics Committee of Tongji Hospital. The protocol was approved by the Human Research Ethics Committee of Tongji Hospital. All subjects gave written informed consent in accordance with the Declaration of Helsinki (LL(H)-09-04) and prospectively registered with the Chinese Clinical Trial Registry^1^ (RN: ChiCTR-TRC-08000732). Eligible participants were randomized into one of three groups: a MDCT, a single-domain cognitive training (SDCT), or a wait list control group. Participants underwent neurocognitive assessment at baseline (pre-intervention), following 24 cognitive training sessions (post-intervention), and at 6-month follow-up. The benefits of MDCT and SDCT on cognitive function are reported elsewhere (Cheng et al., 2012). Among the 270 participants recruited, 193 individuals underwent baseline testing. Among these participants, 165 participants consented to provide blood for genetic testing. Among those who refused to be genotyped, eight were from the MDCT group and eight from the SDCT group. The present study only included participants enrolled in the MDCT and SDCT group to focus the moderating effects of genetic polymorphism on training benefits. Consequently, the final sample size for the current analysis included 104 older adults.

### Cognitive Training Methods

The cognitive training methods are described in detail elsewhere (Cheng et al., 2012). Briefly, training methods were developed based on Gates and Valenzuela’s (2010) operational definition of cognitive training. The training tasks were developed based on several effective tasks documented in the literature (Ball et al., 2002; Noice et al., 2004; Uchida and Kawashima, 2008). The MDCT program included training strategies that tapped into memory function (i.e., episodic memory, face/name associative memory, verbal paired associates learning, semantically unrelated word lists), reasoning (i.e., series of numbers, symbols, words and figures), problem-solving strategies (i.e., Tower of Hanoi), visuospatial map reading skills, handcraft making and physical exercise (e.g., tips on stretching); the SDCT program focused on training reasoning abilities only. Both intervention groups comprised 24 1-h sessions of group training with an average class size of 15 individuals at a frequency of twice a week.

### Cognitive Measures

Seven cognitive domains were measured over three time points. Memory (immediate and delayed recall), visuospatial function, language and attention scores were obtained from the Repeatable Battery for the Assessment of Neuropsychological Status (RBANS; Randolph et al., 1998), which was corrected for age and displayed good reliability and validity in Chinese older adults (Cheng et al., 2011). Cognitive inhibition, executive function and reasoning were measured by the Color-Word Stroop Test (CWST; Boone et al., 1990), the Trails Making Test (TMT; Ashendorf et al., 2008) and the Visual Reasoning Test (VRT; Xiao et al., 2002), respectively.

### Genotyping

The COMT SNP rs4818, and the BDNF SNP rs6265 were selected from ClinVar^2^. Using a Tiangen DNA Isolation Kit (Tiangen Biotech, Beijing, China), leukocyte DNA was isolated from blood samples collected in cubital veins at baseline. SNPs were determined with the TaqMan SNP Genotyping Assay (Applied Biosystems, Foster City, CA, USA) on ABI PRISM 7900 sequence detection system instrument (Applied Biosystems) and SDS 2.0 software (Applied Biosystems). COMT and BDNF SNP distributions showed no deviation from Hardy–Weinberg equilibrium within the entire sample (for COMT, $\chi_{\left(1\right)}^{2}$ = 0.013, *p* = 0.909; for BDNF, $\chi_{\left(1\right)}^{2}$ = 0.634, *p* = 0.426).

### Statistical Analysis

Analyses were performed using both intention-to-treat (ITT) and per protocol (PP) analysis. Missing data points were imputed using Stekhoven and Bühlmann’s missForest method (Stekhoven and Bühlmann, 2012) for the statistical software R (Ihaka and Gentleman, 1996). Multiple comparisons relevant to the analyses mentioned in the following paragraph were corrected using the false discovery rate (FDR) approach in R.

Subsequent analyses were performed using SPSS version 17.0 (SPSS Inc., Chicago, IL, USA). Chi-square or independent *t*-tests were conducted to compare groups across demographic characteristics and baseline cognitive performance. Analyses were also conducted to determine the association between genetic polymorphism and baseline characteristics. To exam the effect of SNPs on cognitive training benefits, a full factorial general linear model (GLM) with repeated measures was conducted which included Time (baseline vs. post-intervention, 6-month follow-up) as the within-group variable, and SNP carrier status (Val/Val vs. Met for BDNF rs6265, and C/C vs. G/- for COMT rs4818, respectively) and Group (MDCT vs. SDCT) as the between-groups variable. All models were adjusted for *a priori* covariates age, gender and education.

## Results

### Participant Characteristics

Among the 104 participants, 50 were randomized to the MDCT group and 54 to the SDCT group. Among those randomized, 45 MDCT participants and 44 SDCT participants completed all three cognitive testing sessions. The sample was 49% female, with a mean age of 70.2 years (SD = 3.7) and a mean education level of 9.6 years (SD = 3.9). The MDCT Group had a larger male-female ratio than the SDCT Group, see Table 1.

**Table 1:** Demographic characteristics and baseline cognitive function by training group.

	MDCT	SDCT	*p*
Gender (male:female)	31:19	22:32	0.030*
Age (years)	70.7 ± 3.5	69.7 ± 3.8	0.155
Education (years)	10.1 ± 3.6	9.1 ± 4.1	0.188
Drop-out	5	10	0.217
RBANS total index	87.0 ± 14.3	86.1 ± 14.5	0.729
Immediate memory	82.7 ± 14.9	80.1 ± 15.6	0.729
Visuospatial/Constructional	99.0 ± 16.0	95.0 ± 16.2	0.392
Language	93.5 ± 12.0	92.1 ± 9.6	0.497
Attention	84.5 ± 17.9	87.2 ± 17.3	0.422
Delayed memory	91.6 ± 15.9	92.9 ± 17.7	0.694
CWST color interfere	20.5 ± 14.1	17.6 ± 9.8	0.227
CWST word interfere	45.5 ± 25.0	39.0 ± 16.8	0.121
TMT A completion time (s)	106.7 ± 48.3	109.2 ± 59.1	0.813
TMT B completion time (s)	202.6 ± 120.3	204.6 ± 107.8	0.926
Visual reasoning test	4.7 ± 2.0	5.0 ± 2.0	0.354

*p < 0.05. MDCT, multi-domain cognitive training; SDCT, single-domain cognitive training; RBANS, Repeatable Battery for the Assessment of Neuropsychological Status; CWST, Color-Word Stroop test; TMT, Trail Making Test.

### Association Between Genetic Polymorphisms and Baseline Characteristics

With respect to polymorphism of the BDNF gene, no statistically significant between groups differences were found, with the exception of cognitive training Group. Specifically, significantly more Val/Val allele carriers were randomized to the SDCT Group relative to the MDCT Group, $\chi_{\left(1\right)}^{2}$ = 4.160, *p* = 0.041. With respect to COMT polymorphism, no statistically significant differences were found, with the exception of sex. Specifically, significantly more males than females were carriers of the COMT C/C allele, $\chi_{\left(1\right)}^{2}$ = 5.126, *p* = 0.024. Further, non-significant trends were found with respect to education and baseline performance on the VRT, with CC carriers of the COMT gene reporting slightly more years of education, *t*_(102)_ = 1.853, *p* = 0.067, and performing slightly better on the VRT, *t*_(102)_ = 1.708, *p* = 0.091, see Table 2.

**Table 2:** Demographic characteristics and baseline cognitive function by genotype group.

	rs6265		*p*	rs4818		*p*
	Val/Val	Met/-		C/C	G/-	
Group (MDCT:SDCT)	8:18	42:36	0.041*	23:17	27:37	0.128
Gender (male:female)	12:14	41:37	0.571	26:14	27:37	0.024*
Age (years)	70.8 ± 3.7	70.0 ± 3.7	0.338	69.6 ± 3.9	70.6 ± 3.6	0.210
Education (years)	9.4 ± 4.1	9.7 ± 3.9	0.729	10.5 ± 3.8	9.1 ± 3.9	0.067
Drop-out	4	11	0.872	6	9	0.895
RBANS total index	85.4 ± 14.5	86.9 ± 14.4	0.652	87.9 ± 15.1	85.7 ± 13.9	0.444
Immediate memory	79.2 ± 17.3	82.1 ± 14.5	0.447	81.4 ± 15.4	81.3 ± 15.2	0.985
Visuospatial/Constructional	99.2 ± 15.7	96.2 ± 16.3	0.415	100.0 ± 16.2	95.0 ± 15.9	0.120
Language	91.3 ± 8.6	93.3 ± 11.4	0.416	93.2 ± 9.9	92.5 ± 11.4	0.763
Attention	84.2 ± 16.3	86.5 ± 18.0	0.577	86.8 ± 16.6	85.3 ± 18.2	0.683
Delayed memory	90.3 ± 19.4	93.0 ± 15.9	0.488	93.2 ± 18.2	91.7 ± 15.9	0.667
CWST color interfere	22.2 ± 9.3	17.9 ± 12.8	0.116	20.7 ± 13.9	17.9 ± 10.8	0.242
CWST word interfere	43.9 ± 21.9	41.6 ± 21.2	0.632	43.6 ± 20.8	41.3 ± 21.7	0.593
TMT A completion time (s)	103.2 ± 38.9	109.6 ± 58.2	0.603	100.3 ± 41.4	112.9 ± 60.3	0.247
TMT B completion time (s)	206.1 ± 80.9	202.8 ± 122.8	0.901	196.6 ± 127.3	208.0 ± 104.6	0.621
Visual reasoning test	4.9 ± 2.1	4.9 ± 1.9	0.954	5.3 ± 2.0	4.6 ± 1.9	0.091

*p < 0.05. MDCT, multi-domain cognitive training; SDCT, single-domain cognitive training; RBANS, Repeatable Battery for the Assessment of Neuropsychological Status; CWST, Color-Word Stroop test; TMT, Trail Making Test.

### The Moderating Role of Genetic Polymorphism on Cognitive Training Outcomes Independent of Training Method

**Figure 1:**
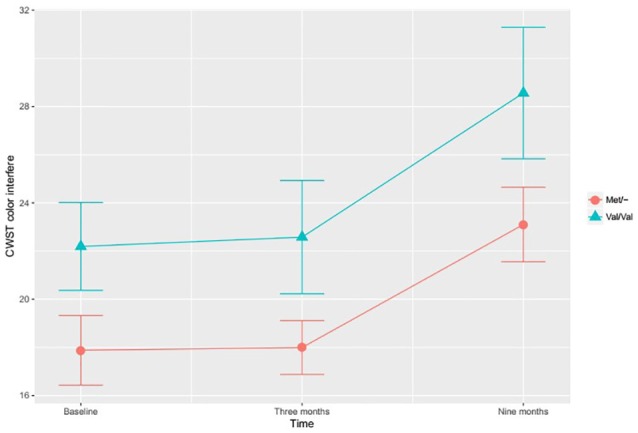
The effects of brain-derived neurotrophic factor (BDNF) polymorphism on inhibition. Met/- carriers performed better than Val/Val carriers on the CWST color interfere task across all time points, *F*_(1,97)_ = 5.417, *p* = 0.022. Note: CWST, Color-Word Stroop test. The error bars represent standard errors.

**Figure 2:**
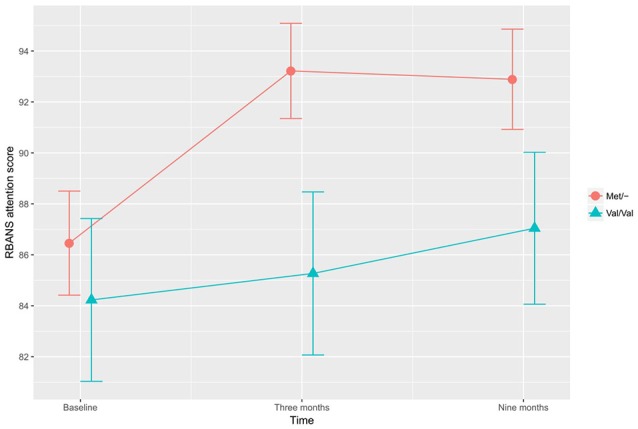
The effects of BDNF polymorphism on attention. Met/- carriers performed better than Val/Val carriers on the RBANS attention tasks across all time points, *F*_(1,97)_ = 3.929, *p* = 0.050. Note: RBANS, Repeatable Battery for the Assessment of Neuropsychological Status. The error bars represent standard errors.

In the investigation of differential training effects based on genetic polymorphism, independent of training mode, no significant associations were found. Instead, the BDNF SNP influenced cognitive function irrespective of cognitive training. According to the adjusted ITT model, a main effect for BDNF SNP was found, with Met/- carriers performing better than Val/Val carriers on the CWST color interfere task, *F*_(1,97)_ = 5.417, *p* = 0.022, Figure 1. This association remained statistically significant in the adjusted PP analysis, *F*_(1,82)_ = 6.519, *p* = 0.013. In the ITT model, adjusting for all *a priori* covariates, the BDNF Met allele was also found to associate with higher RBANS attention score relative to the Val allele, *F*_(1,97)_ = 3.929, *p* = 0.050, Figure 2. This finding remained statistically significant in the adjusted PP analysis, *F*_(1, 82)_ = 4.416, *p* = 0.039.

### The Moderating Role of Genetic Polymorphisms in the Relationship Between Cognitive Training Method and Performance Outcomes

**Figure 3:**
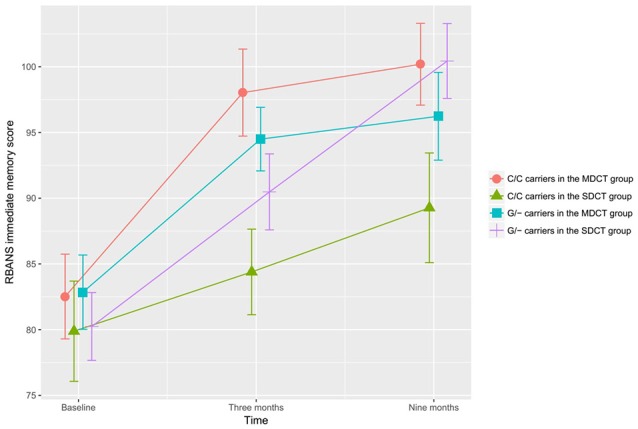
The effects of catechol-O-methyltransferase (COMT) polymorphism on immediate memory. COMT polymorphism determined training benefits in the SDCT group only, indicating a larger gain for G/- carriers than C/C carriers, *F*_(2,104)_ = 4.990, *p* = 0.023, false discovery rate (FDR) corrected. Training benefits differed between MDCT and SDCT in both G/- carriers, *F*_(2,118)_ = 4.624, *p* = 0.023, FDR corrected, and C/C carriers, *F*_(2,70)_ = 4.345, *p* = 0.023, FDR corrected. Note: MDCT, multi-domain cognitive training; SDCT, single-domain cognitive training; RBANS, Repeatable Battery for the Assessment of Neuropsychological Status, respectively. The error bars represent standard errors.

In the ITT adjusted GLM model, a significant Time × Group × COMT interaction was observed for immediate memory, *F*_(2,194)_ = 4.802, *p* = 0.009; Figure 3). Subgroup analyses showed that COMT polymorphism determined training benefits in the SDCT group only (*F*_(2,104)_ = 4.990, *p* = 0.023, FDR corrected), and that training benefits differed between MDCT and SDCT in both C/C (*F*_(2,70)_ = 4.345, *p* = 0.023, FDR corrected) and G/- (*F*_(2, 118)_ = 4.624, *p* = 0.023, FDR corrected) carriers. However, *post hoc* analyses only revealed better performance on the immediate memory task among C/C carriers in the MDCT group compared with C/C carriers in the SDCT group at post-intervention (*M*_MDCT_ = 98.0, *SD* = 15.9 vs. *M*_SDCT_ = 84.4, *SD* = 13.4; *t*_(38)_ = 2.864, *p* = 0.042, FDR corrected). Findings were similar for the adjusted PP model, *F*_(2,164)_ = 5.299, *p* = 0.006. COMT did not moderate any of the other training outcomes. No three-way interactions were observed for BDNF.

## Discussion

Cognitive training has been shown to be a promising remediation tool for cognitive function in late life. However, the underlying mechanisms that predict individual differences in the treatment response remain elusive. The identification of biological parameters may help develop personalized training regimens that compliment biological profiles. This study evaluated the potential moderating role of BDNF and COMT polymorphism on cognitive change following two methods of cognitive training in older Shanghainese. Although COMT polymorphism did not modulate training response across the two training methods, the SNP was found to significantly moderate the association between SDCT and immediate memory performance, with greater gains found among the G/- allele carriers than the C/C carriers. Subsequent *post hoc* tests suggested that C/C carriers benefit more from multi-domain training than single-domain training; whereas this differential effect of training method was not clear in G/- carriers. This result is partially in line with two previous studies, reporting that the Met allele of the functional COMT rs4680 SNP associates with greater transfer effects for working memory than the Val allele following cognitive training (Heinzel et al., 2014; Bellander et al., 2015). Although genetic moderation was found for different tasks (i.e., immediate memory vs. working memory), it may be argued that performance on the RBANS immediate memory index in the current study shares common neural pathways that are required for working memory performance (Cabeza et al., 2002, 2004). Importantly, both rs4680 and rs4818 are located in the central locus of the COMT gene and show linkage disequilibrium in the Chinese population (Xiao et al., 2017).

The current findings contrast with a study conducted in Netherlands (Colzato et al., 2014), which assessed the moderating role of BDNF polymorphism on cognitive function following a 7-week video game play intervention. Specifically, Colzato et al. (2014) reported greater gains in transfer effects for divided attention, but not selective attention, among Val/Val carriers compared with Met/- allele carriers. In the current study, Met/- allele carriers clearly displayed enhanced performance of attention and inhibition compared with the Val/Val carrier group; however, this association was found irrespective of cognitive training. This finding aligns with previous research in older adults suggesting enhanced controlled response and inhibition in Met/- allele carriers compared with Val/Val carriers (Gajewski et al., 2012; Getzmann et al., 2013). BDNF was not found to moderate the training response for memory, visuospatial function, or verbal fluency. With the exception of verbal fluency, memory and visuospatial function are largely hippocampal-dependent (Longoni et al., 2015), which may not be as sensitive to remedial training in healthy older adults as in younger adults (Freundlieb et al., 2012, 2015).

The present findings contribute to a growing body of literature suggesting that genetic polymorphism has the potential to modulate training benefits stemming from cognitive training interventions. Strengths of the current study included evaluating participants from a RCT that employed well-established training patterns for community-dwelling older adults. The study also included measurement of comprehensive cognition domains with a 6-month follow-up period. However, similar to previous studies, the present findings must be interpreted in light of the existing study limitations. First, although the sample size was similar to that reported in previous research, larger randomized trials are needed to validate the current findings and that of previous research. A larger sample will also allow for the exploration of genetic haplotypes and gene-gene interactions on cognitive transfer effects. Indeed, a second limitation of the current study was that the sample size did not allow for statistical evaluation of the combined contributions of COMT and BDNF on cognitive outcomes. Future studies may employ a more informative approach such as haplotypes of tag SNPs or even genome-wide association (GWA). Furthermore, the current sample size did not allow for the investigation of sex effects. Indeed, there is increasing recognition of sex-differentials in brain function and behavioral outcomes (Zhang et al., 2014).

Additional research is required to understand age-differentials in the association between genetic variation and cognitive function, including transfer effects following cognitive training. Much of our understanding about the biological correlates of cognitive function is based on younger individuals, without considering age-related changes in the physiological milieu of the individual. For example, the noted crossover effect in the association between BDNF polymorphism and cognitive function from “young old” to “older old” adults (Erickson et al., 2008) needs further evaluation, which may further help to understand inconsistent research findings. Furthermore, additional research is required to understand how genes correlate with peripheral biomarkers at various stages in the life span and how this may influence cognitive function and transfer effects following cognitive training. Previous research suggests that the observed increase in blood BDNF following cognitive training is restricted to cognitively impaired populations (Vinogradov et al., 2009; Angelucci et al., 2015; Jeong et al., 2016), with no observable changes in blood BDNF (Hakansson et al., 2017; Passaro et al., 2017) or gene expression (Casoli et al., 2014) in healthy older adults. These findings suggest that cognitive training can potentiate substandard levels of BDNF, but may be limited in triggering incremental increases from normal levels.

## Conclusion

This study contributes to a growing body of literature that aims to understand the underlying mechanisms involved in cognitive remediation effects. This line of research is imperative as we move forward in the context of an aging population, with an increasing interest in the creation of biological signatures that may help maximize training benefits to ensure optimal cognitive health in late life.

## Author Contributions

CL designed and supervised the study. YS managed patient recruitment. WF trained the participants. XC, LJ and TL collected the data. JJ and AF performed the data analysis and wrote the first draft of the manuscript. All authors contributed to and have approved the final manuscript.

## Conflict of Interest Statement

The authors declare that the research was conducted in the absence of any commercial or financial relationships that could be construed as a potential conflict of interest.

## Footnotes

^1^http://www.chictr.org.cn

^2^www.ncbi.nlm.nih.gov/clinvar
